# Revealing the nanoparticles aspect ratio in the glass-metal nanocomposites irradiated with femtosecond laser

**DOI:** 10.1038/srep13746

**Published:** 2015-09-08

**Authors:** S. Chervinskii, R. Drevinskas, D. V. Karpov, M. Beresna, A. A. Lipovskii, Yu. P. Svirko, P. G. Kazansky

**Affiliations:** 1 Institute of Photonics, University of Eastern Finland, P.O.Box 111 Joensuu, FI-80101 Finland; 2 Institute of Physics, Nanotechnology and Telecommunications, Peter the Great St. Petersburg Polytechnic University, 29 Polytechnicheskaya, St. Petersburg, 195251 Russia; 3 Optoelectronics Research Centre, University of Southampton, Southampton, SO17 1BJ UK; 4 Department of Physics and Technology of Nanostructures, St. Petersburg Academic University, 8/3 Khlopina, St.-Petersburg, 194021 Russia; 5 Advanced Laser Technologies Centre, Mendeleev University of Chemical Technology of Russia, 9 Miusskaya pl., Moscow, 125047 Russia

## Abstract

We studied a femtosecond laser shaping of silver nanoparticles embedded in soda-lime glass. Comparing experimental absorption spectra with the modeling based on Maxwell Garnett approximation modified for spheroidal inclusions, we obtained the mean aspect ratio of the re-shaped silver nanoparticles as a function of the laser fluence. We demonstrated that under our experimental conditions the spherical shape of silver nanoparticles changed to a prolate spheroid with the aspect ratio as high as 3.5 at the laser fluence of 0.6 J/cm^2^. The developed approach can be employed to control the anisotropy of the glass-metal composites.

Ensembles of elongated metal nanoparticles embedded in transparent media are of interest due to their intrinsic anisotropy^1^ that results in linear and nonlinear dichroism and birefringence^2^,^3^. Conventionally, such anisotropic glass-metal nanocomposites (GMNs) are fabricated by stretching glass slabs containing spherical nanoparticles^4^. In particular, this technique is used in commercially available dichroic polarizers, which employ glasses containing elongated silver or copper nanoparticles. However, modern functional devices require control of the birefringence and dichroism with sub-micron spatial resolution when the shape of the nanoparticles varies over the substrate in a prescribed fashion at distances comparable with the wavelength. These devices are of interest for optical encoding, multidimensional data storage and fabrication of complex, polarization-sensitive spectral masks^5^. The required space-selective shaping of metal nanoparticles can be achieved via irradiating GMNs with intense laser^6^,^7^,^8^,^9^ or ion beams^10^,^11^.

Irradiation of glasses containing spherical silver nanoparticles by femtosecond^12^, picosecond^13^, nanosecond^9^ pulses or even continuous-wave (CW) laser beams^14^ results in the formation of metal spheroids with the processing condition-dependent shape. The type—prolate or oblate—and aspect ratio of the spheroidal nanoparticles are usually characterized with transmission electron microscopy (TEM)^3^,^15^,^16^. TEM and other characterization techniques, however, cannot be used to examine the shape of the nanoparticles *in-situ* as it is required for fabrication of complex, polarization-sensitive planar plasmonic and photonic systems. At the same time, the laser-induced anisotropy of the metal nanoparticles manifests itself in the polarization sensitivity of the optical absorption spectra; these spectra have been thoroughly studied in silver-based GMN in a wide range of expositions^15^. Although the information on the ensemble-averaged aspect ratio of the nanoparticles is stored in the polarization-sensitive absorption and reflection spectra, the problem of revealing the nanoparticles aspect ratio from the linear dichroism of the laser processed GMN is still unresolved.

In this paper, we report an investigation of the linear dichroism spectra of silver-based GMN irradiated with intense femtosecond laser pulses at a wavelength of 515 nm. Using an effective media approximation for ellipsoidal inclusions^17^, we analyze the measured differential transmission spectra for the light polarized parallel (*s*) and perpendicular (*p*) to the polarization azimuth of the femtosecond laser beam used for the GMN modification, and depict the dependence of the aspect ratio of nanoparticles in laser-exposed GMN on the processing conditions.

## Methods

### GMN layer fabrication

GMN samples were prepared from soda-lime float glass by Ag^+^-Na^+^ ion exchange method described in details elsewhere^18^. Ion exchange process was carried out at 325 °C immersing the soda-lime glass slides into Ag_0.5_Na_0.95_NO_3_ solution for 20 minutes, when the subsurface of the glass was enriched with silver ions. Later, the sample was annealed in hydrogen atmosphere at atmospheric pressure for 10 minutes at 250 °C. During the annealing, the silver ions in the glass were reduced and then aggregated into nanoparticles^19^. Finally, we made two roughly 100 nm thick layers of several nanometers sized silver nanoparticles (Ag NPs) embedded in the glass beneath both surfaces of the slide^20^. Our TEM measurements showed that the procedure we use results in step-like concentration profile of Ag NPs in the glass. According to the evaluations from Ref. 20 for similarly manufactured GMN, the volume fraction of spherical nanoparticles is about 0.1 for 2 nm particles radii. This size was taken from the best fit of the measured optical absorption spectrum, supposing the width of the Ag NPs size distribution was below 15%^20^.

### Femtosecond laser modification of glass-silver nanocomposites

**Figure 1:**
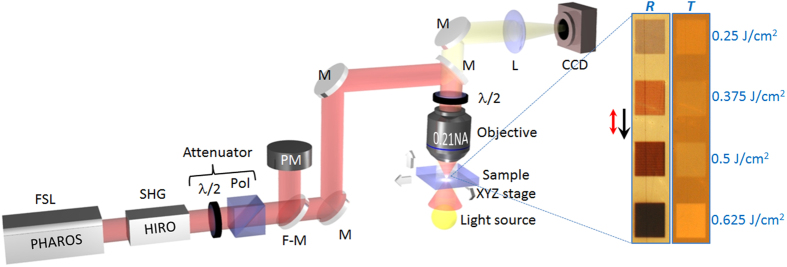
Experimental setup of femtosecond laser modification: femtosecond laser (FSL), second harmonic generator (SHG), half-wave plates (λ/2), polarizer (Pol), power meter (PM), flip-mirror (F-M) allowing to send the radiation to the power meter (PM), dichroic mirrors (M), lens (L), objective lens (0.21 NA), XYZ translation stage. Laser processing was monitored by CCD camera. Inset shows the optical transmission (*T*) and reflection (*R*) images of laser-modified regions of GMN (of 1 × 1 mm^2^) irradiated with various laser fluences. Black arrow indicates the laser writing direction, red arrow—the state of polarization. Drawn by Rokas Drevinskas.

In order to investigate the laser modification of the GMN, the sample was irradiated with 330 fs pulses generated by regeneratively amplified, mode-locked Yb:KGW based ultrafast laser system (Pharos, Light Conversion Ltd.) operating at 515 nm (frequency doubled) at a 20 kHz repetition rate. We irradiated the series of 1 × 1 mm^2^ square regions of the sample by writing 1 mm lines with 2 μm interline distance. The writing speed was 0.5 mm/s, and the laser pulse energy was controlled by a half-wave plate and linear polarizer (Fig. 1) and varied in the range of 0.01–0.065 μJ for different squares. The laser beam was polarized parallel to the writing direction. The beam was focused inside the substrate via a 0.21 NA objective lens providing a net fluence of 0.25–1.625 J/cm^2^ (0.76–4.9 TW/cm^2^), each point under the beam was irradiated with ~60 laser pulses. For a set of laser energies the series of squares were written (Inset in Fig. 1).

### Laser induced linear dichroism measurements

**Figure 2:**
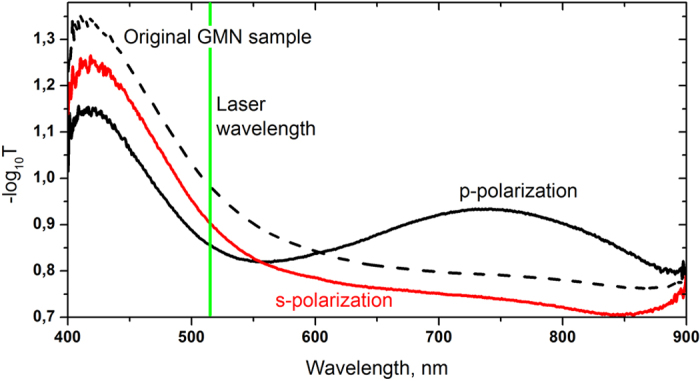
Absorbance spectra of original GMN (dashed), and after laser irradiation with 0.5 J/cm^2^ fluence (solid) in *s* (red) in *p* (black) polarized light. Green line denotes the wavelength of the modifying laser.

Optical characterization of the pristine and irradiated GMN regions was performed with UV-VIS-NIR microspectrometer system (Olympus BX51, CRAIC). The absorbance spectrum of the pristine GMN was measured using a non-polarized light (Fig. 2). The dichroism of the irradiated regions of the GMN was studied by measuring differential optical density 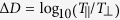, where 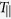 and 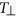 are the transmittances of light polarized along (*s*-polarization) and perpendicular (*p*-polarization) to the writing beam polarization, respectively (Fig. 2). We controlled the polarization of the probe light beam by inserting a linear polarizer before the sample.

## Results

**Figure 3:**
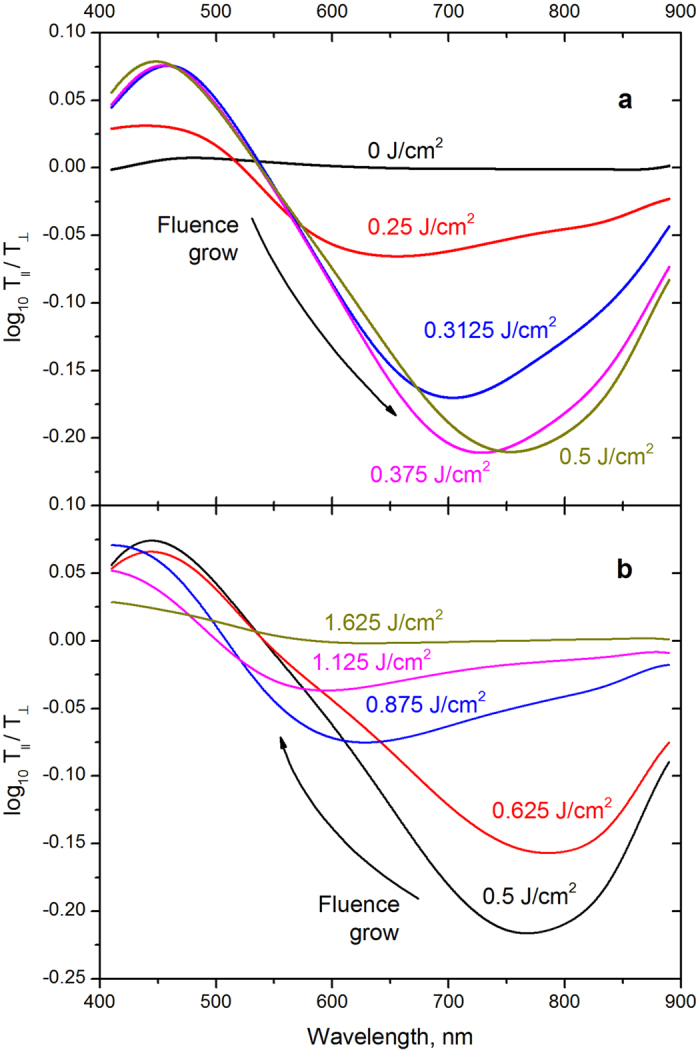
Differential optical density of the modified GMN. (**a**) Low power regime: the resonance band shifts towards longer wavelengths, and its strength increases as the laser power increases. (**b**) High-power regime: the resonance band shifts towards shorter wavelengths, and its strength decreases as the laser power increases.

Figure 3 demonstrates the difference in optical densities 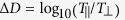
of the modified GMN for orthogonally polarized probe beams. One can see two strong bands in the vicinity of 450 nm and 750 nm that dominate the linear dichroism spectrum of the processed GMN substrate. The linear dichroism originates from the splitting of the surface plasmon resonance (SPR) when spherical silver nanoparticles are elongated by the femtosecond laser irradiation^2^. In other words, the SPR position in the transmittance spectrum is different for *s* and *p*-polarized probe beams.

**Figure 4:**
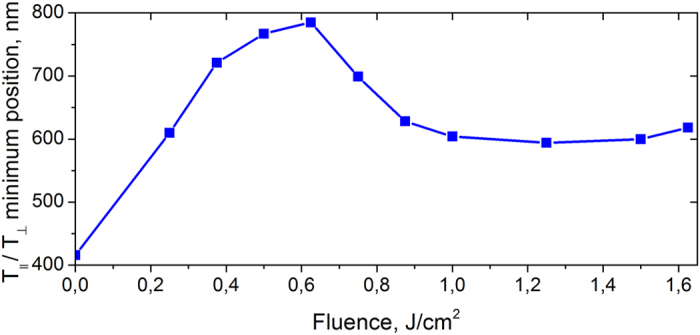
The position of the differential optical density minimum as a function of laser fluence. The data are extracted from the spectra shown in Fig. 3.

Figure 3 shows that the linear dichroism of the modified GMN is non-monotonous function of the laser fluence. Specifically, the SPR associated with 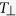 (i.e. when ΔD is negative) becomes stronger when the fluence increases up to 0.625 J/cm^2^ (Fig.3(a)), and its strength decreases for higher fluences (Fig. 3(b)). Figure 4 shows the spectral position of the differential optical density minimum as a function of laser fluence. The dependence behaviour can be due to the heat accumulation at higher laser intensities that prevents complete solidification of silver spheroids between two subsequent femtosecond pulses and partial destruction of nanoparticles^15^.

## Discussion

The reshaping of silver nanoparticles under laser irradiation is caused by the enhancement of the local electric field in the vicinity of metal inclusion in dielectric. At high laser intensities, the local field gives rise to the ejection of electrons from metal nanoparticle into the glass matrix and drastically increases the local temperature^15^ driving the system out of equilibrium. This provokes the transformation of spherical nanoparticles to spheroids with rotation axis along the polarization azimuth of the laser beam and eventually leads to the optical dichroism.

For the analysis of the GMN modification, it is instructive to consider optical properties of the homogeneous composite medium consisting of identical metal spheroids with permittivity 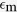, embedded into a host matrix with permittivity 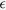. We assume that the spheroids size is much less than light wavelength and that they all have the same shape and orientation prescribed by the polarization of the processing femtosecond laser beam. Since we studied silver-based GMN with the metal concentration less than 15 vol.%, we employ Maxwell Garnett approach (MGA)^21^ by describing the permittivity tensor of the composite in terms of the polarizability tensor of the spheroids^1^.

The polarizability tensor of an isolated spheroid with the radii of *a* and *c* (rotation axis) can be presented in the following form: *α*_*ij*_ = *v*_0_*β*_*ij*_, where *v*_0_ = 4*πca*^2^/3 is the volume of the spheroid. If *z* axis is directed along the rotation axis *c* of the spheroid, tensor *β*_*ij*_ is diagonal one, 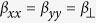 and 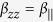, and^1^


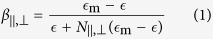


Here 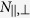 are depolarization factors of the spheroid^1^,


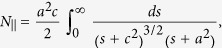


and 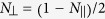. For a sphere 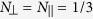, while for prolate and oblate spheroids the depolarization factor is 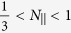 and 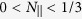, respectively.

**Figure 5:**
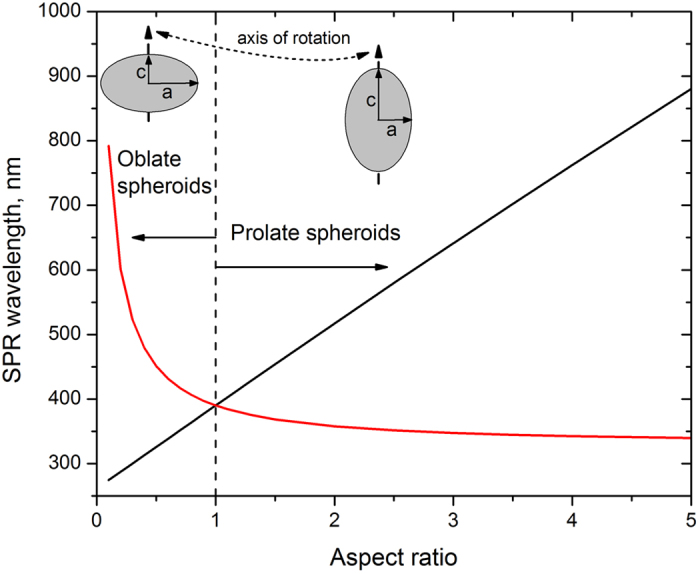
Surface plasmon resonance wavelengths calculated for oblate and prolate silver spheroids of different aspect ratios in the glass matrix. Red and black solid line show SPR wavelength for the light polarized along *a*- and *c*-axis, respectively. The following parameters were used for the numerical simulations: 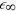 =4, *λ*_*p*_ = 135 *nm*, *γ*/*ω*_*p*_ = 0.1[23], 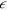 = 2.72.

The dielectric permittivity of silver in the visible spectral range can be well described in the Drude model framework^22^:


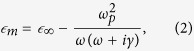


where 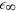 is the high frequency permittivity, *ω*_*p*_ is the plasma frequency, and *γ* is the electron scattering rate^23^. The model^1^ allows calculating SPR for a silver spheroid for the plasmonic oscillation along *a*-axis and *c*-axis. The dependence of SPR wavelengths on the spheroid aspect ratio is illustrated in Fig. 5. It is worth noting that in Fig. 5, the electron scattering at the surface of the spheroid is not taken into consideration, i.e. the size of the spheroid is not accounted for.

When the *c*-axes of the spheroids are aligned in the same direction, the composite possesses the properties of uniaxial material. Thus the dielectric permittivity tensor of the composite is diagonal, 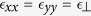 and 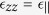, where^17^


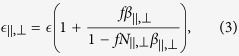


and *f* is the volume fraction of the spheroidal inclusions.

It is convenient to express 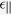 and 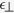 in terms of 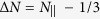, a quantitative measure of the spheroidicity of nanoparticles that constitute the composite:


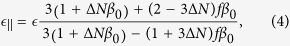



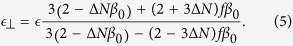


Here


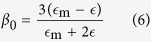


represents the polarizability *α*_0_ = *v*_0_*β*_0_ of a sphere with volume *v*_0_. One can see that at Δ*N* = 0, Eqs (4,5) give the permittivity of the isotropic composite consisting of spherical nanoparticles, 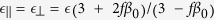. When Δ*N* ≠ 0, i.e. when nanoparticles are spheroids, the GMN is anisotropic. However, the optical anisotropy of the composite can be controlled not only by the particle spheroidicity Δ*N*, but also by the volume fraction *f*. In particular, Eqs (3,4) yield 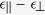 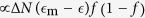, i.e. in the Maxwell Garnett composite (*f* ≪ 1), the anisotropy is proportional to the product of Δ*Nf*. This implies that the change of particles shape and metal volume fraction provides us two independent channels for the GMN optical response control.

The anisotropy of the composite comprising of metal spheroids embedded into the dielectric matrix manifests itself in the linear dichroism contrary to almost pure birefringence observed in glasses without nanoparticles after ultrashort pulse irradiation^24^. Specifically, in the irradiated nanocomposite the position and the width of the SPR are different for the light polarized along *c*- and *a*-axis of the spheroid. The SPR wavelength in a spherical particle can be determined within the conditions of vanishing the real part of the denominator in Eq. (6), 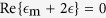. However, in the composites comprised of spheroids, the SPR resonances for the light polarized along *c*- and *a*-axes take place at different frequencies that can be found from the following equations:


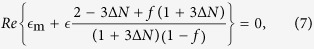



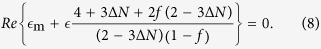


It is instructive to obtain an analytical formula for the SPR resonance in the composite using Drude model. By using Eqs (7,8) and taking into account that in the visible spectral range *ω*_*p*_ ≫ *γ*, one can obtain the following equations for the surface plasmon wavelengths for light polarized along and perpendicular rotation axis of metal spheroids comprising the composite:


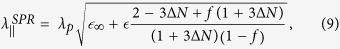



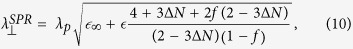


where *λ*_*p*_ = 2*πc/ω*_*p*_ is the plasma wavelength. At 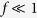, 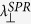 and 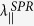 depend on the aspect ratio similarly to resonant wavelengths of the isolated spheroid (see Fig. 5).

By using Eqs (9,10) we can show that with *ω*_*p*_, *ω* ≫ *γ*, the SPR has Lorentzian shape,


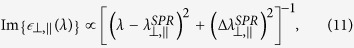


where the linewidth is determined by the electron scattering rate,


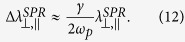


It is necessary to mention that electron scattering rate *γ* in a nanoparticle can be considerably higher than that in a bulk metal. Specifically, for silver spherical nanoparticle with radius *r*, the scattering rate is *γ* = 1/*τ* + *AV*_*F*_/*r* where *τ* ≅ 30 fs and *V*_*F*_ = 1.4 × 10^8^ cm/s are the electron collision time and Fermi velocity in the bulk silver, respectively, and *A* is the parameter of the order of one^23^. Since in our experiment, the GMN was comprised of silver nanoparticles with radius less than 10 nm^22^, one may expect that in our experimental conditions, the collisions of free electrons with the particle’s surface dominates *γ*.

From Eqs (9,10) we observe that SPR can be shifted by changing the metal volume fraction. In particular, for prolate spheroids 0 < Δ*N *< 2/3, 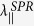 varies in the spectral region 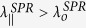 while 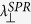 changes in the region 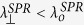. Here


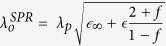


is the SPR wavelength for the nanocomposite comprised of spherical nanoparticles.

It is worth noting that modification of the particle shape also affects the electron scattering rate *γ* in Eq. (8). Specifically, in spheroids, the collision rate, which is the function of radius in spherical metal nanoparticles^23^, depends on whether electron moves along *c-* or *a-*axis^25^. If we assume that the particle volume *v*_0_ = 4*πr*^3^/3 does not change under its transformation from a sphere to a spheroid, the scattering rates for electrons oscillating along *c-* and *a-* axis can be expressed as 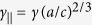 and 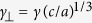, respectively.

Equations (4,5) allow us to model polarized transmission spectra of the structure consisted of a layer of the modified GMN, bare glass substrate, and a layer of the pristine GMN comprising spherical nanoparticles. The latter should be taken into account because the ion exchange technique (see Section 2) results in the formation of nanoparticles beneath both surfaces of the glass plate, while the tight focusing of the femtosecond laser beam leaves the back surface of the sample unmodified.

**Figure 6:**
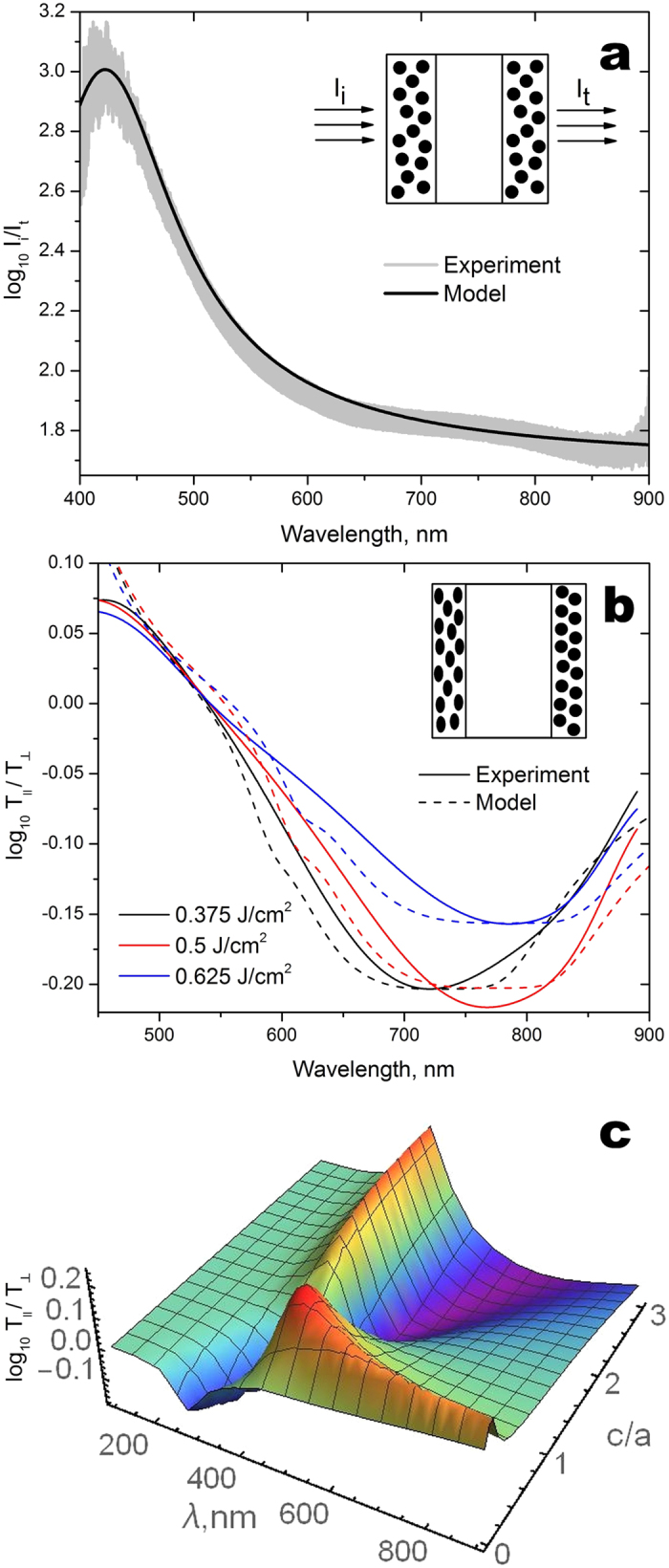
(**a**) Absorbance of the non-modified GMN calculated using MGA (black solid line) and obtained in the experiment (wide grey line). (**b**) Solid lines represent differential optical density of the GMN after laser irradiation with fluence of 0.375 J/cm^2^ (black), 0.5 J/cm^2^ (red), and 0.625 J/cm^2^ (blue). Dash lines represent differential optical density of the GMN calculated using MGA upon *c/a* = 3.32, *ξ* = 0.37 (black); *c*/*a* = 3.61, *ξ* = 0.38 (red), and *c*/*a* = 3.74, *ξ* = 0.31 (blue). Inset shows nanoparticles inside the laser modified sample. (**с**) Simulated spectrum of the differential optical density vs aspect ratio and resonance wavelength for *ξ* = 0.31. The following parameters were used for the numerical simulations: 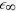 =4, *λ*_*p*_ =135 nm, *γ*/*ω*_*p*_ = 0.09, *L* = 10 nm, *f* = 0.06, *α*_*s*_ = 0.016 nm^−1^, the refractive indices of the bare and silver-enriched glass are 1.5 and 1.65, respectively.

In order to reveal the parameters of nanocomposite we performed fitting of measured linear absorption spectrum of the non-modified GMN. In the fitting, we used conventional Drude model parameters for silver, 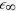 = 4, *λ*_*p*_ = 135 *nm*, *γ*/*ω*_*p*_ = 0.09^23^, and assumed that the refractive indices of the bare and silver-enriched glass are 1.5 and 1.65. This difference in the refractive indices originates both from the ion exchange and increase of silver ions concentration in the subsurface region of glasses in the course of the hydrogen processing^19^,^20^. The scattering losses in the subsurface layer were taken into account phenomenologically by introducing extinction coefficient *α*_*s*_ = 0.0016 *nm*^−1^. One can observe from Fig. 6(a) that we obtain a good agreement between the calculated (solid line) and measured (broad grey line) spectra upon the *f *= 0.06 and the thickness of silver-enriched layer *L* = 100 nm.

The obtained parameters of the non-modified composite allow us to reproduce the spectra measured after the femtosecond laser irradiation (Section 2.2). We assume that the laser processing results in the transformation of spherical metal nanoparticles into spheroids only beneath the front surface of the sample. However, it is worth noting that in our experiments, the distance between lines is 2 μm, with the spot diameter of 1.6 μm. According to the lower intensity in the periphery of the focal spot and the periodicity of the inscribed lines, considerable area of the processed region remains unmodified. Despite the fact that only modified regions of the sample possess linear dichroism, both modified and non-modified areas contribute to the transmittance: 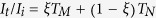. Here *T*_*M*_ and *T*_*N*_ are transmittances of the modified and non-modified regions, while *ξ* describes relative area of the modified region. That is from Fig. 6(b), where we presented experimental and calculated spectra of differential optical density 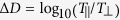, one may conclude that in our experimental conditions more than 30% of the nanoparticles beneath the front surface of the glass were modified. We also simulated the spectrum of the differential optical density as a function of the spheroids aspect ratio and plot it in coordinates of *c*/*a* and resonance wavelength (Fig. 6(c)).

**Figure 7:**
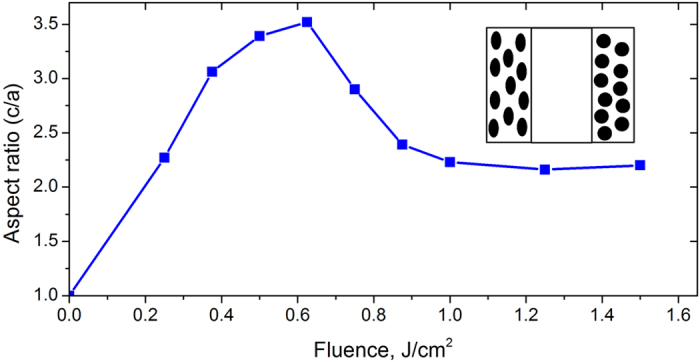
Aspect ratio, *c/a*, of prolate silver spheroids as a function of the modification laser fluence. The relation between the peaks of differential optical density and the aspect ratio of spheroids were extracted from the processed transmission spectra partly shown in Figs 3 and 6(b). The inset schematically show nanoparticles shape and location inside the laser irradiated sample.

The fitting of the experimental spectra shown in Fig. 6(b) allows us to obtain the aspect ratio of reshaped particles as a function of the fluence of the processing laser beam (Fig. 7). By comparing Figs 4 and 7, one can conclude that 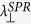 is a nearly linear function of the aspect ratio the same way as for isolated spheroids in Fig. 5. In our experimental conditions, this function takes the following form: λ_SPR_[nm] ≈ 145(*c*/*a*) + 275 nm. This is in a good agreement with the estimations made for the isolated spheroids^26^.

One can observe from Fig. 7 that in our experiment, we obtained the maximum aspect ratio of *c/a *≈ 3.5 at the fluence of about 0.625 J/cm^2^. It is worth noting however that increasing laser fluence results in the drop of the aspect ratio down to ~2.6. The observed phenomenon can be understood if one recall that elongation of nanoparticles within focal area of the writing beam is accompanied by their rapid heating due to the relatively slow (0.5 mm/s) translation of the sample and relatively high (20 kHz) repetition rate. Since each pulse increases the nanoparticle temperature by 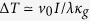, where *I* is the laser pulse intensity, *k*_*g*_ ≈ 1 *W*/*mK* is the thermal conductivity of the glass, *v*_0_ is the nanoparticle volume^27^, even at moderate intensities melting of the nanoparticle and softening the surrounding glass occur^28^. These thermal effects influence the electric and surface tension forces, whose balance determines the shape of the nanoparticle. In our experimental conditions, one may expect the elongation of the nanoparticles caused by electrical forces at fluences above 0.625 J/cm^2^ results in their partition with the formation of less elongated nanoparticles, which gives rise to the decreasing of aspect ratio at fluences between 0.625 J/cm^2^ and 1 J/cm^2^. When we increase fluence higher a strong local electric field eventually destroys nanoparticles^15^,^29^ similarly to their destruction by strong DC electric field^30^.

## Conclusion

By comparing the experimental and theoretical differential transmittance spectra of the femtosecond laser processed silver-based GMN we demonstrated that in the wide range of the laser fluences the aspect ratio of the reshaped silver nanoparticles comprising GMN is a linear function of the fluence. This result opens avenues towards the control of shape and optical properties of metal nanoparticles essential for a variety of plasmonic and nanophotonic applications. In particular, this is important for modification of GMN with high spatial resolution in order to create a plasmon enhanced gratings and widely tunable surface components. Spectral separation between the SPR peaks of orthogonally polarized light as high as 400 nm was obtained, that corresponds to transformation of the spherical silver nanoparticles into prolate spheroids with the aspect ratio up to 3.5. This light-induced anisotropy can be increased even further by using GMN substrates with larger metal particles and by controlling the overall modification process.

## Additional Information

**How to cite this article**: Chervinskii, S. *et al.* Revealing the nanoparticles aspect ratio in the glass-metal nanocomposites irradiated with femtosecond laser. *Sci. Rep.* **5**, 13746; doi: 10.1038/srep13746 (2015).
