# Corrigendum: Revealing the nanoparticles aspect ratio in the glass-metal nanocomposites irradiated with femtosecond laser

**DOI:** 10.1038/srep18522

**Published:** 2016-01-27

**Authors:** S. Chervinskii, R. Drevinskas, D. V. Karpov, M. Beresna, A. A. Lipovskii, Yu. P. Svirko, P. G. Kazansky

Scientific Reports 5: Article number: 13746; 10.1038/srep13746published online 09082015; updated: 01272016

The Acknowledgements section in this Article is incomplete.

“The study has been supported by EU (Marie Curie FP7 project #269140), Academy of Finland (grant #288151 and #265015), EPSRC (grant EP/M029042/1), and Russian Science Foundation (grant no. 14-22-00136). P.G.K. thanks Ministry of Education and Science of the Russian Federation (Grant No. 14.Z50.31.0009). Authors are grateful to Amin Abdolvand for valuable comments on the manuscript.”

should read:

“The study has been supported by EU (Marie Curie FP7 project #269140), Academy of Finland (grant #288151 and #265015), EPSRC (grant EP/M029042/1), and Russian Science Foundation (grant no. 14-22-00136). P.G.K. thanks Ministry of Education and Science of the Russian Federation (Grant No. 14.Z50.31.0009). Authors are grateful to Amin Abdolvand for valuable comments on the manuscript. The data for this work is accessible through the University of Southampton Institutional Research Repository (http://dx.doi.org/10.5258/SOTON/384152).”

